# Overcoming acquired resistance of EGFR‐mutant NSCLC cells to the third generation EGFR inhibitor, osimertinib, with the natural product honokiol

**DOI:** 10.1002/1878-0261.12645

**Published:** 2020-02-14

**Authors:** Hongjing Zang, Guoqing Qian, Jack Arbiser, Taofeek K. Owonikoko, Suresh S. Ramalingam, Songqing Fan, Shi‐Yong Sun

**Affiliations:** ^1^ Department of Pathology The Second Xiangya Hospital Central South University Changsha China; ^2^ Department of Hematology and Medical Oncology Emory University School of Medicine and Winship Cancer Institute Atlanta GA USA; ^3^ Department of Dermatology Emory University School of Medicine and Winship Cancer Institute Atlanta Veterans Administration Medical Center Atlanta GA USA

**Keywords:** acquired resistance, apoptosis, EGFR, honokiol, lung cancer, osimertinib

## Abstract

The development of acquired resistance to osimertinib (Osim) (AZD9291 or TAGRISSO^TM^), an FDA‐approved third‐generation epidermal growth factor receptor (EGFR) inhibitor for the treatment of EGFR‐mutant nonsmall cell lung cancer (NSCLC), limits the long‐term benefits for patients. Thus, effective treatment options are urgently needed. To this end, we explored whether honokiol (HNK), a natural product with potential antitumor activity, may be used to overcome Osim resistance. The combination of HNK and Osim synergistically decreased the survival of several Osim ‐resistant cell lines with enhanced effects on inhibiting cell colony formation and growth and on inducing apoptosis. This combination also showed greater growth suppression of Osim‐resistant xenograft tumors including those with 19del, T790M, and C797S triple mutations in nude mice. Mechanistically, the augmented induction of apoptosis by the combination is largely due to enhanced Mcl‐1 reduction through facilitating its degradation. A synthetic HNK derivative exerted similar effects with greater efficacy. Our findings warrant further study of HNK and its derivatives in overcoming Osim resistance in the clinic.

## Graphical abstract

The development of acquired resistance to osimertinib (Osim), an FDA‐approved third‐generation epidermal growth factor receptor (EGFR) inhibitor for the treatment of EGFR‐mutant nonsmall cell lung cancer, limits the long‐term benefits for patients. The natural product, honokiol, when combined with Osim, augmented induction of apoptosis in different Osim ‐resistant cell lines primarily through enhancing Mcl‐1 degradation and effectively inhibited the growth of Osim‐resistant tumors.

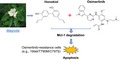

**Abbreviations**
CHX: cycloheximide
EGFR: epidermal growth factor receptor
EGFR‐TKIs: EGFR‐tyrosine kinase inhibitors
HNK: honokiol
KO: knockout
NSCLC: nonsmall cell lung cancer
Osim: Osimertinib

## 1 Introduction

Lung cancer is the leading cause of cancer death worldwide and thus remains a serious health issue and economic burden (Torre *et al.*, 2016). In recent years, targeted therapies have transformed the treatment of lung cancer patients with a specific mutation. Among them, epidermal growth factor receptor‐tyrosine kinase inhibitors (EGFR‐TKIs) benefit lung cancer patients with activating EGFR mutations (Tartarone and Lerose, 2015). This group of drugs has been developed rapidly from the initial 1st generation (e.g., gefitinib and erlotinib) to 2nd generation (e.g., afatinib) and now 3rd generation [e.g., osimertinib (Osim); also named AZD9291 or TAGRISSO™] EGFR‐TKIs (Russo *et al.*, 2017). However, the emergence of acquired resistance in patients occurs more rapidly than new‐generation agents can be developed, and eventual treatment failure is inevitable (Russo *et al.*, 2017).

The appearance of the T790M resistance mutation is the major mechanism accounting for ~ 60% of relapse cases from 1‐st generation EGFR‐TKI treatment (Juchum *et al.*, 2015; Remon *et al.*, 2014; Tartarone and Lerose, 2015). To overcome this mutation, Osim and other 3‐rd generation EGFR‐TKIs were developed, which selectively and irreversibly inhibit EGFR carrying the common ‘sensitive’ mutations, 19del and L858R, and the resistant T790M mutation without affecting wild‐type EGFR. Osim is an FDA‐approved drug for the treatment of NSCLC harboring activating EGFR mutations (first‐line) or that has become resistant to 1‐st generation EGFR‐TKIs through the T790M mutation (second‐line). Without exception, patients also develop resistance to Osim, preventing long‐term clinic remission (Govindan, 2015; Ramalingam *et al.*, 2017). Hence, the development of effective strategies to overcome resistance to Osim and other 3‐rd generation EGFR‐TKIs is very important in the clinic setting.

Honokiol (HNK) is a natural product purified from the Magnolia tree and has been used in Chinese, Korean, and Japanese traditional medicine (Banik *et al.*, 2019; Lee *et al.*, 2011). Many preclinical studies have demonstrated the potential antitumor activity of HNK against different types of cancer such as breast cancer, neuroblastoma, bladder cancer, pancreatic cancer, and lung cancer (Averett *et al.*, 2016; Hsiao *et al.*, 2019; Lin *et al.*, 2019; Pan *et al.*, 2017; Sengupta *et al.*, 2017). With the goal of developing efficacious combinatorial regimens to overcome Osim resistance, we have identified HNK as a potential agent that may sensitize Osim‐resistant NSCLC cells to Osim. Thus, this study focused on demonstrating the activity of HNK and its derivatives, when combined with Osim, against the growth of different Osim‐resistant cell lines *in* *vitro* and *in vivo* and understanding the underlying mechanisms.

## 2 Materials and methods

### 2.1 Reagents

The source and preparation of Osim and cycloheximide (CHX) were the same as described previously (Shi *et al.*, 2017). HNK and its derivatives diethylstilbestrol (DEtS) and carbazole p‐isomer (CAz‐p) were described previously (Raja *et al.*, 2008). Mcl‐1, PARP, p‐AKT (S473), AKT, p‐ERK1/2 (T202/Y204), and ERK1/2 antibodies were purchased from Cell Signaling Technology, Inc (Beverly, MA, USA). Bim antibody was purchased from EMD Millipore (Burlington, MA, USA). GAPDH antibody was purchased from Proteintech, Inc (Rocky Hill, NJ, USA).

### 2.2 Cell lines and cell culture

The Osim ‐resistant cell lines PC‐9/AR, PC‐9/GR/AR, PC‐9/3M, and HCC827/AR and culture conditions were the same as described previously (Shi *et al.*, 2017). The PC‐9 cell line with EGFR 19del and C797S double mutation (named PC‐9/2M herein) used in a previous study (Niederst *et al.*, 2015) was kindly provided by A. N. Hata (Harvard Medical School, Boston, MA, USA). H1975/OSIR was provided by Y. Yang (Mayo Clinic, Rochester, MN, USA). The features of these cell lines are summarized in Table 1. The PC‐9/3M/Mcl‐1 stable cell line (pooled populations) was generated by infecting PC‐9/3M cells with lentiviruses carrying ectopic Mcl‐1 or vector for 48 h followed by selection using zeocin (500 µg·mL^−1^) for another 7 days as described previously (Ren *et al.*, 2013). PC‐9/GR/AR/Bim‐KO cell lines were generated by infecting cells simultaneously with lentiviruses carrying CRISPR/Cas9 and SgBim for 48 h followed by puromycin selection for another 5 days as described previously (Qian *et al*. 2018). Single‐cell clones were picked for screening for Bim knockout (KO) using western blotting.

**Table 1:** Osim‐resistant EGFR‐mutant NSCLC cell lines used in this study.

Resistant cell line	EGFR mutations	MET alteration	Isogenic parental line
First‐line Osim resistance			
HCC827/AR	19del	Amplification	HCC827
PC‐9/AR	19del	None	PC‐9
PC‐9/2M	19del, C797S (trans)	None	PC‐9
H1975/OSIR	L858R, T790M	None	H1975
Second‐line Osim resistance			
PC‐9/GR/AR	19del, T790M	None	PC‐9/GR
PC‐9/3M	19del, T790M, C797S (cis)	None	PC‐9

### 2.3 Cell survival and apoptosis assays

Cells seeded in 96‐well plates were treated the second day with the tested agents. After a given time period of treatment, viable cells were determined using sulforhodamine B (SRB) assay as described previously (Sun *et al.*, 1997). The calculation of combination index for drug interaction (e.g., synergy) was performed using compusyn software (Combo Syn, Inc., Paramus, NJ, USA). Cell apoptosis was detected with an annexin V/7‐AAD apoptosis detection kit (BD Biosciences, San Jose, CA, USA) following the manufacturer’s manual. PARP was detected by western blotting as another indicator of apoptosis.

### 2.4 Western blot analysis

The procedures for the preparation of whole‐cell protein lysates and western blot analysis were the same as we described previously (Shi *et al.*, 2017; Shi *et al.*, 2016).

### 2.5 Colony formation assay

The effects of the tested treatments on colony formation were conducted in 12‐well plates as previously described (Shi *et al.*, 2017; Shi *et al.*, 2016).

### 2.6 Protein stability assay

Mcl‐1 protein stability was determined using CHX chase assay as described previously (Koo *et al.*, 2015; Shi *et al.*, 2017).

### 2.7 Animal xenograft and treatments

Animal experiments, which were approved by the Institutional Animal Care and Use Committee (IACUC) of Emory University, were carried out as described previously (Shi *et al.*, 2017). The treatments included vehicle control, Osim (10 mg·kg^−1^·day^−1^, 5 days·week^−1^, og), HNK (50 mg·kg^−1^·day^−1^; ip), and their combination. We shared vehicle and Osim treatments with another study that examined the activity of LBH589 and Osim combination against Osim ‐resistant tumors (Li *et al.*, 2019) in order to minimize the usage of animals. Tumor volumes were measured using caliper measurements and calculated with the formula *V* = (length × width^2^)/2. At the end of the treatment, mice were weighed and euthanized with CO_2_ asphyxia. The tumors were then collected and frozen in liquid nitrogen after weighting.

### 2.8 Statistical analysis

The statistical significance of differences between two groups was analyzed with two‐sided unpaired Student's *t*‐tests when the variances were equal. Data were examined as suggested by the same software to verify that the assumptions for use of the *t*‐tests held. Differences among multiple groups were analyzed with one‐way ANOVA. *P* values < 0.05 were considered to be statistically significant.

## 3 Results

### 3.1 Combination of HNK and osimertinib synergistically decreases the survival of varied EGFR‐mutant NSCLC cell lines with acquired resistance to Osim and inhibits colony formation and growth

**Figure 1:**
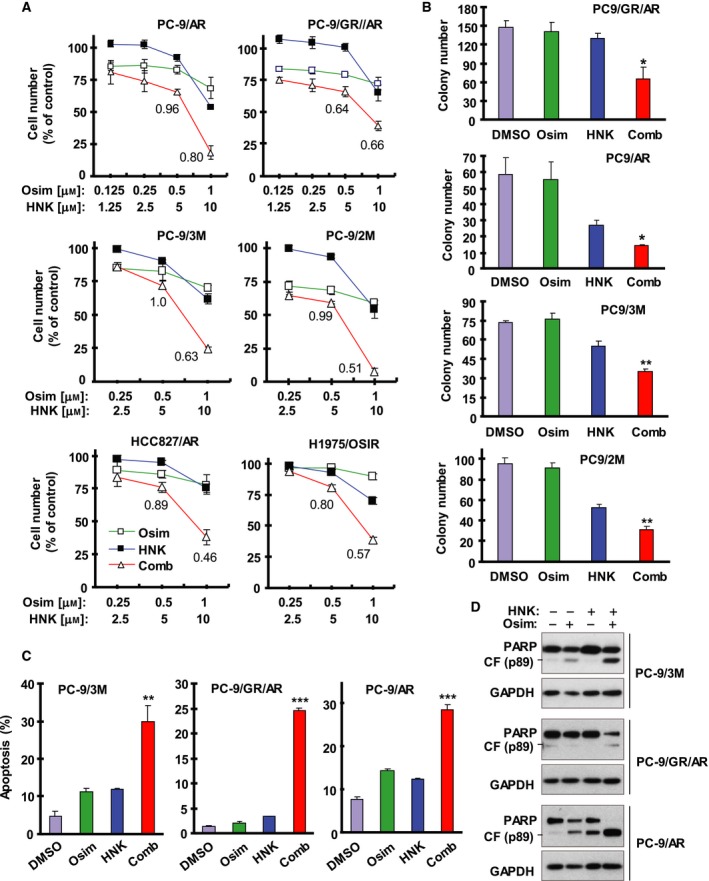
The combination of HNK and Osim synergistically decreases the survival and inhibits colony formation and growth of Osim‐resistant EGFR‐mutant NSCLC cell lines with augmented induction of apoptosis. (A) The indicated cell lines seeded in 96‐well plates were treated the next day with the given concentrations of Osim alone, HNK alone, or their combinations. After 72 h, cell numbers were estimated using the SRB assay. The numbers inside the graphs are CIs for the given combinations. (B) The indicated cell lines were seeded in 12‐well cell culture plates. On the second day, the cells were treated with fresh medium containing DMSO, 5 µm HNK alone, 200 nm Osim alone, and HNK plus Osim and the treatment was repeated every 3 days for a total of 12 days. (C, D) The indicated cell lines were exposed to DMSO, 10 µm HNK, 1 µm Osim, or HNK plus Osim for 72 h (C) or 48 h (D) and then harvested for the detection of apoptosis with annexin V/flow cytometry (C) and for the detection of PARP cleavage with western blotting (D). The data are means ± SDs of four replicates (A), triplicate (B), or duplicate (C) determinations. *P < 0.05, **P < 0.01, and ***P < 0.001 at least compared with other treatments. CF, cleaved form.

We first determined the effect of Osim in the presence of HNK on the growth of different Osim ‐resistant EGFR‐mutant NSCLC cell lines including PC‐9/AR, PC‐9‐GR/AR, PC‐9/2M (19del and C797S are trans located), PC‐9/3M (19del, T790M, and C797S are cis located), HCC827/AR, and H1975/OSIR (Table 1). The combination of HNK and Osim was more active than either agent alone in decreasing the survival of these cell lines. The CIs were < 1, particularly when HNK at 10 µm was used, indicating synergistic effects on decreasing cell survival (Fig. 1A). The long‐term colony formation assay also showed that the combination of HNK and Osim was more effective than either single agent alone in suppressing the formation and growth of colonies in several Osim ‐resistant cell lines (Fig. 1B). Clearly, the presence of HNK is able to resensitize these Osim‐resistant cell lines to Osim.

### 3.2 Combination of HNK and osimertinib enhances induction of apoptosis in osimertinib‐resistant cell lines

We next determined whether the combination effectively decreased the survival of these resistant cell lines through enhancing induction of apoptosis. As evaluated with annexin V/flow cytometry, the combination of HNK and Osim significantly induced apoptosis in comparison with each agent alone, which induced little or no apoptosis in PC‐9/AR, PC‐9/GR/AR, and PC‐9/3M cells (Fig. 1C). Similarly, we detected much greater levels of cleaved PARP in all three tested cell lines exposed to the combination of HNK and Osim than in cells treated with either agent alone. The levels of intact or uncleaved PARP were also reduced much more in PC‐9/GA/AR and PC‐9/3M cells exposed to the combination than in cells treated with either single agent alone (Fig. 1D). Collectively, these results demonstrate that the combination of HNK and Osim enhances induction of apoptosis in Osim ‐resistant cell lines.

### 3.3 Combination of HNK and osimertinib enhances reduction in Mcl‐1 levels through facilitating its degradation in osimertinib‐resistant cells

**Figure 2:**
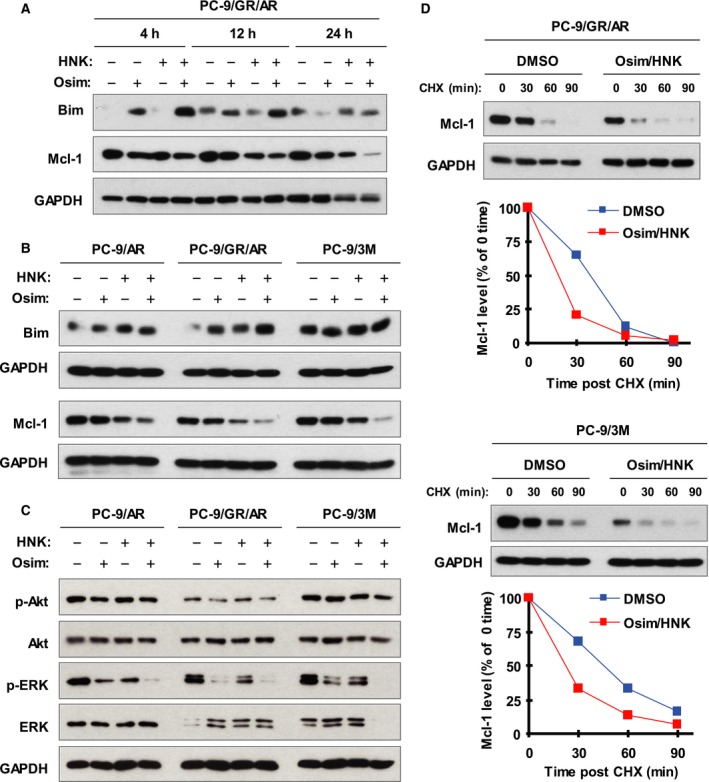
The combination of HNK and Osim enhances the reduction in Mcl‐1 levels accompanied by augmented suppression of ERK through facilitating Mcl‐1 degradation in Osim‐resistant cells. (A‐C) The indicated cell lines were exposed to 1 µm Osim alone, 5 µm HNK alone, or their combination for the given times (A) or 12 h (B and C) and then harvested for the preparation of whole‐cell protein lysates and subsequent western blot analysis. (D) The given cell lines were treated with DMSO or 5 µm HNK plus 1 µm Osim for 8 h followed by the addition of 10 µg·mL^−1^ CHX. At the indicated times post‐CHX, the cells were harvested for the preparation of whole‐cell protein lysates and subsequent western blot analysis. Band intensities were quantified by NIH Image J software, and Mcl‐1 levels were presented as a percentage of levels at 0 time post‐CHX treatment.

To understand the molecular mechanism by which the HNK and AZD9291 combination augments induction of apoptosis, we examined its effects on the levels of Bim and Mcl‐1, two key molecules involved in mediating apoptosis induced by Osim in sensitive EGFR‐mutant NSCLC cells as we recently demonstrated (Shi *et al.*, 2017). In PC‐9/GR/AR cells, the combination of HNK and Osim was clearly more potent than each agent alone in increasing Bim levels and in decreasing Mcl‐1 levels across the tested time period from 4 to 24 h (Fig. 2A). In two additional Osim‐resistant cell lines, PC‐9/AR and PC‐9/3M, the combination also enhanced the reduction in Mcl‐1 levels although the enhancement of Bim elevation was not observed after 12‐h treatment (Fig. 2B). Collectively, it is clear that the combination of HNK and Osim universally enhances the reduction in Mcl‐1 levels in Osim‐resistant cell lines accompanied by enhanced Bim elevation in some cell lines.

We assessed the effects of the HNK and Osim combination on Akt and ERK phosphorylation in these resistant cell lines. The combination had no enhanced effects on p‐Akt levels, but clearly augmented the decrease in p‐ERK1/2 levels in comparison with single‐agent treatment across the tested cell lines (Fig. 2C).

Since p‐ERK suppression is associated with enhanced Mcl‐1 degradation induced by Osim in sensitive EGFR‐mutant NSCLC cells, as demonstrated recently (Shi *et al*. 2017), we then logically asked whether the HNK and Osim combination facilitates Mcl‐1 degradation in Osim‐resistant cells. By performing CHX assays, we observed that Mcl‐1 was degraded more rapidly in both PC‐9/GA/AR and PC‐9/2M cell lines exposed to the HNK and AZD9291 combination than in those exposed to DMSO control (Fig. 2D), suggesting that the combination of HNK and Osim indeed enhances Mcl‐1 degradation in Osim‐resistant cells.

### 3.4 Enforced overexpression of Mcl‐1 protects AZD9291‐resistant cells from being killed by the combination of HNK and osimertinib

**Figure 3:**
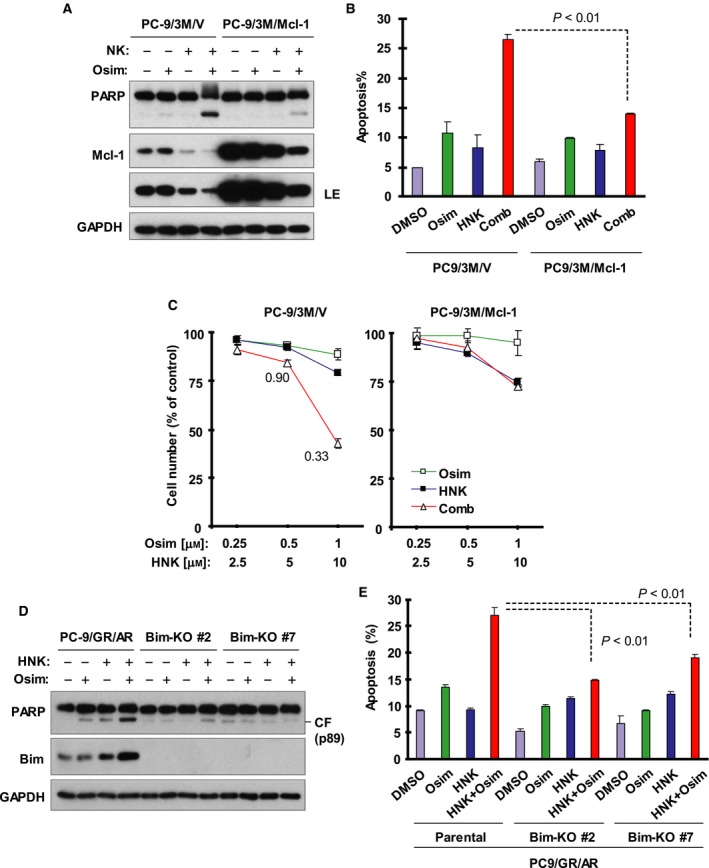
Enforced overexpression of ectopic Mcl‐1 or Bim‐KO protects AZD9291‐resistant cells from being killed by the combination of HNK and Osim as evaluated by apoptosis and cell survival. (A, B, D, and E) The given cell lines with different levels of Mcl‐1 (A) or deficient in Bim (D) were exposed to 1 µm Osim alone, 10 µm HNK alone, or their combination for 48 h and then harvested for the measurement of apoptotic cells with annexin V/flow cytometry (B and E) and for the detection of PARP cleavage using western blotting (A and D). The data in B and E are means ± SDs of duplicate determinations. LE, longer exposure. (C) The indicated cell lines seeded in 96‐well plates were treated with different concentrations of HNK alone, Osim alone, and their concentrations as indicated for 72 h. Cell numbers were estimated with the SRB assay. The data are means ± SDs of four replicate determinations.

To determine the involvement of Mcl‐1 reduction in mediating induction of apoptosis by the HNK and Osim combination, we introduced an Mcl‐1 expression construct into PC‐9/3M cells through lentiviral infection to generate the cell line PC‐9/3M/Mcl‐1 that expressed ectopic Mcl‐1 as we confirmed with western blotting (Fig. 3A). This cell line was significantly less sensitive than the vector control cell line (PC‐9/3M/V) to undergo apoptosis induced by the HNK and Osim combination as evidenced by reduced PARP cleavage (Fig. 3A) and limited increase in annexin V‐positive cells (Fig. 3B). In agreement, the combination of HNK and Osim synergistically decreased the survival of PC‐9/3M/V cells, but failed to do so in PC‐9/3M/Mcl‐1 cells (Fig. 3C). Collectively, these data demonstrate that enforced overexpression of ectopic Mcl‐1 attenuates the ability of the HNK and Osim combination to induce apoptosis and death of Osim‐resistant cells.

### 3.5 Bim knockout in PC‐9/GA/AR cells confers resistance to the combination of HNK and osimertinib

Since in PC‐9/GR/AR cells the combination of HNK and Osim clearly enhanced the levels of Bim, another critical molecule in mediating Osim‐induced apoptosis in sensitive EGFR‐mutant NSCLC cells (Shi *et al.*, 2017), we determined whether enhanced Bim elevation contributes to the augmented induction of apoptosis by the HNK and Osim combination. We used the CRISPR/Cas9 technique to knock out Bim in PC‐9/GR/AR cells (Fig. 3D) and then assessed their responses to treatment with HNK and Osim. Both PC‐9/GR/AR/Bim‐KO#2 and PC‐9/GR/AR/Bim‐KO#7 cells were much less sensitive than their parental PC‐9/GA/AR cells to undergoing apoptosis upon co‐treatment with HNK and Osim as evaluated with both PARP cleavage (Fig. 3D) and annexin V staining (Fig. 3E). Thus, Bim deficiency in PC‐9/GA/AR cells confers resistance to the combination of HNK and Osim.

### 3.6 The combination of HNK and osimertinib enhances the growth suppression of osimertinib‐resistant xenografts in nude mice

**Figure 4:**
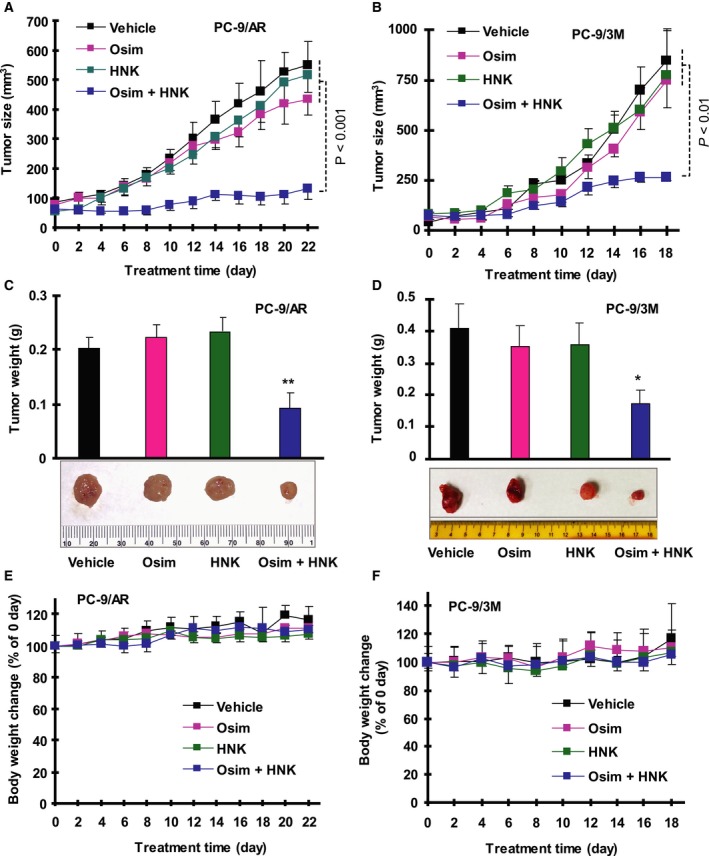
The combination of HNK and Osim effectively inhibits the growth of PC‐9/AR and PC‐9/3M xenografts *in vivo*. Both PC‐9/AR and PC‐9/3M xenografts were treated (once a day for 5 days·week^−1^) with vehicle control, Osim, HNK, and their combination starting on the same day after grouping. Tumor sizes (A, B) were measured as indicated. Each measurement is mean ± SE (*n* = 6). At the end of the treatment, the mice were sacrificed to remove tumors, which were weighed (C, D). Mouse body weights were also compared (E, F). *P < 0.05 at least compared with all other groups; **P < 0.01 at least compared with all other groups.

Following these *in vitro* studies, we then used xenograft models in nude mice to determine whether the combination of HNK and Osim has enhanced inhibitory effects against the growth of Osim‐resistant tumors *in vivo*. In agreement with our observations in cell cultures described above, the combination of HNK and Osim significantly inhibited the growth of both PC‐9/AR (Fig. 4A,C) and PC‐9/3M (Fig. 4B,D) xenografts in comparison with vehicle control, HNK alone, or Osim alone as evaluated by measurement of both tumor size (Figs 4A,B) and weight (Fig. 4C,D). Under the tested conditions, both HNK and Osim alone did not significantly inhibit tumor growth (Fig. 4A–D). Moreover, the combination did not apparently decrease mouse body weights over the tested period (Fig. 4E,F), suggesting that the combination was well‐tolerated by mice.

### 3.7 The HNK derivative, CAz‐p, exerts similar effects in synergizing with osimertinib against osimertinib‐resistant cells with increased potency

**Figure 5:**
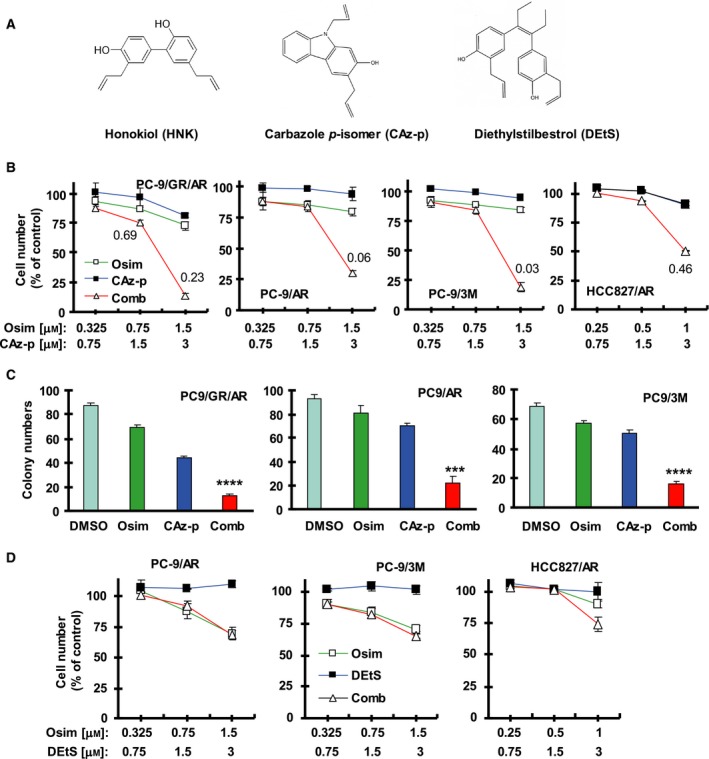
The HNK derivatives CAz‐p and DEtS have distinctive effects in decreasing the survival and inhibiting colony formation and growth of Osim ‐resistant EGFR‐mutant NSCLC cell lines when combined with Osim. (A) Chemical structures of HNK derivates tested in this study. (B, D) The indicated cell lines seeded in 96‐well plates were treated the next day with the given concentrations of AZD9291 alone, CAz‐p or DEtS alone, or their combinations. After 72 h, cell numbers were estimated using the SRB assay. The numbers inside the graphs are CIs for the given combinations. (C) The indicated cell lines were seeded in 12‐well cell culture plates. On the second day, the cells were treated with fresh medium containing DMSO, 1 µm (PC‐9/AR and PC‐9/GR/AR) or 2 µm (PC‐9/3M) CAz‐p alone, 200 nm Osim alone, and CAz‐p plus Osim and the treatment was repeated every 3 days for a total of 12 days. The data are means ± SDs of four replicates (B and D) or triplicate (C) determinations. ***P < 0.001 and ****P < 0.0001 at least compared with other treatments.

Two HNK derivatives, DEtS and CAz‐p (Fig. 5A), showed greater activities than HNK in suppressing growth and inducing apoptosis of lung cancer cells as demonstrated in our previous study (Raja *et al.*, 2008). We thus examined the effects of these derivatives in combination with Osim on the growth of Osim‐resistant cells. The combination of CAz‐p (e.g., 3 µm) and Osim (e.g., 1.5 µm) was clearly much more potent than either agent alone in decreasing the survival of four Osim‐resistant cell lines with CIs far smaller than 1 (Fig. 5B), suggesting synergistic effects. Similar results were also generated in the colony formation assay (Fig. 5C). However, we found that DEtS combined with Osim, under the same tested conditions, did not enhance the reduction in survival of the tested Osim‐resistant cell lines compared with single‐agent treatment (Fig. 5D).

**Figure 6:**
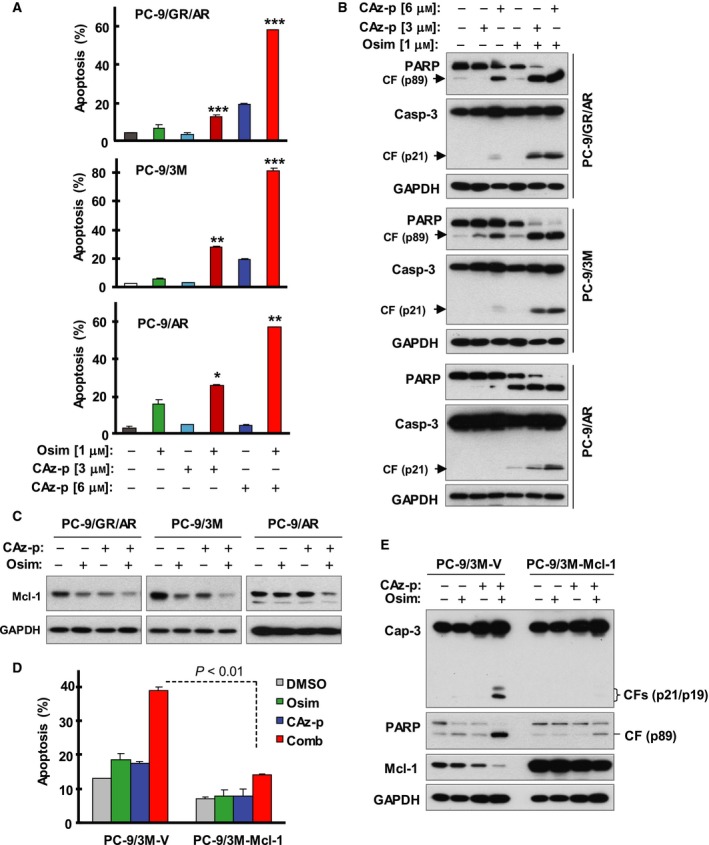
The combination of CAz‐P and Osim enhances the induction of apoptosis in Osim ‐resistant EGFR‐mutant NSCLC cell lines through downregulation of Mcl‐1. (A,B) The indicated cell lines were exposed to DMSO, 3 or 6 µm CAz‐p, 1 µm Osim, or CAz‐p plus Osim for 48 h (PC‐9/AR) or 72 h (PC‐9/GR/AR and PC‐9/3M) and then harvested for the detection of apoptosis with annexin V/flow cytometry (A) and for the detection of PARP cleavage with western blotting (B). (C) The indicated cell lines were treated with DMSO, 1 µm Osim, 6 µm CAz‐p, or CAz‐p plus Osim for 12 h. The proteins of interest were detected using western blotting. (D, E) The given cell lines were exposed to DMSO, 1 µm Osim, 3 µm CAz‐p, or CAz‐p plus Osim for 48 h (E) or 72 h (D) and harvested for measuring apoptosis with annexin V/flow cytometry (D) and for the detection of protein cleavage with western blotting (E). The data in A and D are means ± SDs of duplicate determinations. *P < 0.05, **P < 0.01, and ***P < 0.001 at least compared with other treatments.

We further analyzed the effects of CAz‐p combined with Osim on the induction of apoptosis in three Osim‐resistant cell lines and found that CAz‐p at 3 µm and particularly at 6 µm, when combined with Osim, was significantly more effective than either agent alone in inducing apoptosis of PC‐9/AR, PC‐9/GR/AR, and PC‐9/3M cell lines as evaluated by measuring annexin V‐positive cells (Fig. 6A) and by detecting the cleavage of caspase‐3 and PARP (Fig. 6B). Moreover, the combination of CAz‐p with Osim also effectively enhanced Mcl‐1 reduction in these cell lines (Fig. 6C). Enforced expression of ectopic Mcl‐1 in PC‐9/3M cells significantly compromised the effect of the CAz‐p and Osim combination on induction of apoptosis, as indicated by comparing both annexin V‐positive cells (Fig. 6D) and caspase‐3 and PARP cleavage (Fig. 6E). These findings suggest a critical role of Mcl‐1 reduction in mediating the enhanced induction of apoptosis in Osim‐resistant cells induced by the CAz‐p and Osim combination.

## 4 Discussion

The current study provides both *in vitro* and *in vivo* evidence demonstrating that the natural product, HNK, when combined with Osim, effectively inhibits the growth of Osim‐resistant cells and tumors. Hence, HNK has the potential to overcome acquired resistance to Osim. The appearance of the C797S resistant mutation is now a defined mechanism for the emergence of acquired resistance to Osim, which accounts for 20–30% of resistant cases when Osim is used as a second‐line treatment (Murtuza *et al.*, 2019). Currently, there are no effective options for the treatment of resistant tumors with triple mutations of EGFR at 19del, T790M, and C797S. The intriguing finding in our study is that the HNK and Osim combination effectively decreased the survival and induced apoptosis of both PC‐9/2M cells harboring trans EGFR 19del and C797S double mutations and PC‐9/3M cells carrying cis EGFR 19del, T790M, and C797S triple mutations. Another advantage of HNK is that it is a natural product used as a nutritional supplement in humans and thus has good tolerability and safety (Sarrica *et al.*, 2018). Approximately 33% of patients with EGFR‐mutant NSCLC develop brain metastasis (Franchino *et al.*, 2018), which is the main cause of mortality in this population. While Osim has improved efficacy against brain metastases (Le and Gerber, 2019), another valuable feature of HNK is that it can readily cross the blood–brain barrier and the blood–cerebrospinal fluid barrier (Wang *et al.*, 2011; Woodbury *et al.*, 2013), making it a suitable candidate for combining with Osim; this speculation is worthy of further investigation. Collectively, our findings warrant future evaluation of the potential of HNK to overcome Osim acquired resistance in the clinic.

The combination of HNK and Osim augmented apoptosis in the tested Osim ‐resistant cell lines. This enhanced induction of apoptosis is likely an important mechanism contributing to the enhanced antitumor activity of the combination of HNK and Osim against Osim‐resistant cells and tumors. We have recently demonstrated that Mcl‐1 reduction and Bim elevation are key mechanisms mediating Osim‐induced apoptosis in sensitive EGFR‐mutant NSCLC cells (Shi *et al.*, 2017). Although enhanced Bim levels were detected only in one of three tested Osim‐resistant cell lines (i.e., PC‐9/GR/AR), augmented reduction in Mcl‐1 was observed across the tested Osim‐resistant cell lines including PC‐9/AR, PC‐9/GR/AR, and PC‐9/3M when exposed to the HNK and Osim combination. These findings suggest a more critical role for Mcl‐1 reduction than Bim elevation in mediating the enhanced induction of apoptosis induced by the HNK and Osim combination. This is further supported by our data showing that Mcl‐1 overexpression protects Osim‐resistant cells from apoptosis induced by HNK and Osim co‐treatment. Since Bim‐KO in PC‐9/GR/AP cells also protected the cells from undergoing apoptosis induced by the combination of HNK and Osim, it is fair to conclude that enhanced Bim elevation may also contribute to augmented induction of apoptosis by HNK and Osim combination at least in some Osim‐resistant cell lines (e.g., PC‐9/GR/AR). A recent study has shown that the natural product, bufalin, can reverse acquired resistance to Osim through induction of Ku70‐mediated Mcl‐1 degradation (Cao *et al.*, 2019). Moreover, it has been shown that EGFR‐mutant NSCLC cells tolerated to short‐term EGFR‐TKI treatment possess elevated Mcl‐1 levels and can be sensitized to EGFR‐TKIs by targeting Mcl‐1 (Song *et al.*, 2018). These studies together with our findings here thus highlight critical involvement of Mcl‐1 in the development of acquired resistance to EGFR‐TKIs including Osim and suggest a possible strategy for delaying and/or overcoming acquired resistance to Osim by co‐targeting Mcl‐1. *Bim* deletion polymorphism, which occurs in East Asians at a frequency of 21% but is rare in African and European populations, has been associated with Osim resistance (Li *et al.*, 2018; Tanimoto *et al.*, 2017). Given the dominant role of Mcl‐1 suppression in mediating induction of apoptosis by Osim and HNK combination in various Osim‐resistant cell lines, it is plausible to speculate that this combination may be effective against the resistance to Osim due to *Bim* deletion polymorphism. Hence, further study in this direction is warranted.

ERK phosphorylates Mcl‐1 protein, resulting in its stabilization (Domina *et al.*, 2004; Nifoussi *et al.*, 2012). Osim inhibits ERK‐dependent Mcl‐1 phosphorylation and facilitates Mcl‐1 degradation, leading to Mcl‐1 reduction in sensitive EGFR‐mutant NSCLC cells, as we have recently demonstrated (Shi *et al.*, 2017). We found that the combination of HNK and Osim indeed enhanced Mcl‐1 degradation. Since enhanced Mcl‐1 reduction was accompanied by augmented ERK suppression (i.e., suppression of ERK phosphorylation) in the tested Osim ‐resistant cell lines, it is plausible to suggest that the HNK and Osim combination enhances Mcl‐1 reduction or degradation likely through augmented suppression of ERK‐dependent Mcl‐1 phosphorylation.

One potential drawback or concern regarding the use of natural products is their relatively weak biological activities. In this study, we found that CAz‐p, a derivative of HNK, exerted similar effects as HNK did in decreasing the survival and enhancing the induction of apoptosis of several Osim ‐resistant cell lines including PC‐9/3M, showing potential in overcoming Osim acquired resistance. CAz‐p achieved these effects at a concentration of 3 µm, which is threefold lower than that required for HNK to exert similar effects (e.g., 10 µm), indicating that CAz‐p has greater potency than HNK in overcoming acquired resistance to Osim. Therefore, it is possible to use HNK and CAz‐p as lead compounds for developing novel agents with optimized efficacy and pharmacological characteristics to overcome Osim acquired resistance.

## 5 Conclusions

The current study has identified HNK and its derivatives as potential candidates for overcoming acquired resistance to Osim caused by varied mechanisms such as C797S mutation. Our findings provide strong preclinical support for testing this potential strategy for overcoming acquired Osim resistance in the clinic and warrant further studies to fully understand the underlying molecular mechanisms and to develop HNK derivatives that can overcome Osim acquired resistance.

## Conflict of interest

SSR is on consulting/advisory board for AstraZeneca, BMS, Merck, Roche, Tesaro, and Amgen. TKO is on consulting/advisory board for Novartis, Celgene, Lilly, Sandoz, Abbvie, Eisai, Takeda, Bristol‐Myers Squibb, MedImmune, Amgen, AstraZeneca, and Boehringer Ingelheim. Other people declare that they have no conflict of interest.

## Author contributions

HZ and GQ designed and conducted experiments as well as contributed to the writing of the paper; SF contributed to the concept and paper writing; JA provided HNK and its derivatives and participated in discussion of the project; TWO and SSR contributed to the concept and paper writing; and SSY directly supervised the study, designed experiments, and wrote the paper.

## References

[mol212645-bib-0001] Averett C , Bhardwaj A , Arora S , Srivastava SK , Khan MA , Ahmad A , Singh S , Carter JE , Khushman M and Singh AP (2016) Honokiol suppresses pancreatic tumor growth, metastasis and desmoplasia by interfering with tumor‐stromal cross‐talk. Carcinogenesis 37, 1052–1061. 27609457 10.1093/carcin/bgw096 PMC5091041

[mol212645-bib-0002] Banik K , Ranaware AM , Deshpande V , Nalawade SP , Padmavathi G , Bordoloi D , Sailo BL , Shanmugam MK , Fan L , Arfuso F *et al* (2019) Honokiol for cancer therapeutics: a traditional medicine that can modulate multiple oncogenic targets. Pharmacol Res 144, 192–209. 31002949 10.1016/j.phrs.2019.04.004

[mol212645-bib-0003] Cao F , Gong YB , Kang XH , Lu ZH , Wang Y , Zhao KL , Miao ZH , Liao MJ and Xu ZY (2019) Degradation of MCL‐1 by bufalin reverses acquired resistance to osimertinib in EGFR‐mutant lung cancer. Toxicol Appl Pharmacol 379, 114662. 31301315 10.1016/j.taap.2019.114662

[mol212645-bib-0004] Domina AM , Vrana JA , Gregory MA , Hann SR and Craig RW (2004) MCL1 is phosphorylated in the PEST region and stabilized upon ERK activation in viable cells, and at additional sites with cytotoxic okadaic acid or taxol. Oncogene 23, 5301–5315. 15241487 10.1038/sj.onc.1207692

[mol212645-bib-0005] Franchino F , Ruda R and Soffietti R (2018) Mechanisms and therapy for cancer metastasis to the brain. Front Oncol 8, 161. 29881714 10.3389/fonc.2018.00161 PMC5976742

[mol212645-bib-0006] Govindan R (2015) Overcoming resistance to targeted therapy for lung cancer. N Engl J Med 372, 1760–1761. 25923556 10.1056/NEJMe1500181

[mol212645-bib-0007] Hsiao CH , Yao CJ , Lai GM , Lee LM , Whang‐Peng J and Shih PH (2019) Honokiol induces apoptotic cell death by oxidative burst and mitochondrial hyperpolarization of bladder cancer cells. Exp Ther Med 17, 4213–4222. 30988795 10.3892/etm.2019.7419 PMC6447899

[mol212645-bib-0008] Juchum M , Gunther M and Laufer SA (2015) Fighting cancer drug resistance: opportunities and challenges for mutation‐specific EGFR inhibitors. Drug Resist Updat 20, 10–28. 10.1016/j.drup.2015.05.002 26021435

[mol212645-bib-0009] Koo J , Yue P , Deng X , Khuri FR and Sun SY (2015) mTOR complex 2 stabilizes Mcl‐1 protein by suppressing its GSK3‐dependent and SCF‐FBXW7‐mediated degradation. Mol Cell Biol 35, 2344–2355. 25918246 10.1128/MCB.01525-14 PMC4456440

[mol212645-bib-0010] Le T and Gerber DE (2019) Newer‐generation EGFR inhibitors in lung cancer: how are they best Used? Cancers 11, 366. 10.3390/cancers11030366 PMC6468595 30875928

[mol212645-bib-0011] Lee YJ , Lee YM , Lee CK , Jung JK , Han SB and Hong JT (2011) Therapeutic applications of compounds in the Magnolia family. Pharmacol Ther 130, 157–176. 21277893 10.1016/j.pharmthera.2011.01.010

[mol212645-bib-0012] Li X , Wang S , Li B , Wang Z , Shang S , Shao Y , Sun X and Wang L (2018) BIM deletion polymorphism confers resistance to osimertinib in EGFR T790M lung cancer: a case report and literature review. Target Oncol 13, 517–523. 29907952 10.1007/s11523-018-0573-2

[mol212645-bib-0013] Li Y , Zang H , Qian G , Owonikoko TK , Ramalingam SR and Sun SY (2019) ERK inhibition effectively overcomes acquired resistance of epidermal growth factor receptor‐mutant non‐small‐cell lung cancer cells to osimertinib. Cancer. [Epud ahead of prints]. 10.1002/cncr.32655 PMC7050394 31821539

[mol212645-bib-0014] Lin MC , Lee YW , Tseng YY , Lin YW , Chen JT , Liu SH and Chen RM (2019) Honokiol Induces autophagic apoptosis in neuroblastoma cells through a P53‐dependent pathway. Am J Chin Med 47, 895–912. 31091975 10.1142/S0192415X19500472

[mol212645-bib-0015] Murtuza A , Bulbul A , Shen JP , Keshavarzian P , Woodward BD , Lopez‐Diaz FJ , Lippman SM and Husain H (2019) Novel third‐generation EGFR tyrosine kinase inhibitors and strategies to overcome therapeutic resistance in lung cancer. Cancer Res 79, 689–698. 30718357 10.1158/0008-5472.CAN-18-1281

[mol212645-bib-0016] Niederst MJ , Hu H , Mulvey HE , Lockerman EL , Garcia AR , Piotrowska Z , Sequist LV and Engelman JA (2015) The allelic context of the C797S mutation acquired upon treatment with third generation EGFR inhibitors impacts sensitivity to subsequent treatment strategies. Clin Cancer Res 21, 3924–3933. 25964297 10.1158/1078-0432.CCR-15-0560 PMC4587765

[mol212645-bib-0017] Nifoussi SK , Vrana JA , Domina AM , De Biasio A , Gui J , Gregory MA , Hann SR and Craig RW (2012) Thr 163 phosphorylation causes Mcl‐1 stabilization when degradation is independent of the adjacent GSK3‐targeted phosphodegron, promoting drug resistance in cancer. PLoS ONE 7, e47060. 23056582 10.1371/journal.pone.0047060 PMC3467206

[mol212645-bib-0018] Pan J , Lee Y , Zhang Q , Xiong D , Wan TC , Wang Y and You M (2017) Honokiol decreases lung cancer metastasis through inhibition of the STAT3 signaling pathway. Cancer Prev Res 10, 133–141. 10.1158/1940-6207.CAPR-16-0129 PMC6005650 27849557

[mol212645-bib-0019] Qian G , Yao W , Zhang S , Bajpai R , Hall WD , Shanmugam M , Lonial S and Sun SY (2018) Co-inhibition of BET and proteasome enhances ER stress and Bim-dependent apoptosis with augmented cancer therapeutic efficacy. Cancer Lett 434, 44–54. 10.1016/j.canlet.2018.07.033 30059709

[mol212645-bib-0020] Raja SM , Chen S , Yue P , Acker TM , Lefkove B , Arbiser JL , Khuri FR and Sun SY (2008) The natural product honokiol preferentially inhibits cellular FLICE‐inhibitory protein and augments death receptor‐induced apoptosis. Mol Cancer Ther 7, 2212–2223. 18645030 10.1158/1535-7163.MCT-07-2409 PMC2756752

[mol212645-bib-0021] Ramalingam SS , Yang JC , Lee CK , Kurata T , Kim DW , John T , Nogami N , Ohe Y , Mann H , Rukazenkov Y *et al* (2017) Osimertinib as first‐line treatment of EGFR mutation‐positive advanced non‐small‐cell lung cancer. J Clin Oncol 36, 841–849. 28841389 10.1200/JCO.2017.74.7576

[mol212645-bib-0022] Remon J , Moran T , Majem M , Reguart N , Dalmau E , Marquez‐Medina D and Lianes P (2014) Acquired resistance to epidermal growth factor receptor tyrosine kinase inhibitors in EGFR‐mutant non‐small cell lung cancer: a new era begins. Cancer Treat Rev 40, 93–101. 23829935 10.1016/j.ctrv.2013.06.002

[mol212645-bib-0023] Ren H , Zhao L , Li Y , Yue P , Deng X , Owonikoko TK , Chen M , Khuri FR and Sun SY (2013) The PI3 kinase inhibitor NVP‐BKM120 induces GSK3/FBXW7‐dependent Mcl‐1 degradation, contributing to induction of apoptosis and enhancement of TRAIL‐induced apoptosis. Cancer Lett 338, 229–238. 23562472 10.1016/j.canlet.2013.03.032 PMC3750077

[mol212645-bib-0024] Russo A , Franchina T , Ricciardi GRR , Smiroldo V , Picciotto M , Zanghi M , Rolfo C and Adamo V (2017) Third generation EGFR TKIs in EGFR‐mutated NSCLC: where are we now and where are we going. Crit Rev Oncol Hematol 117, 38–47. 28807234 10.1016/j.critrevonc.2017.07.003

[mol212645-bib-0025] Sarrica A , Kirika N , Romeo M , Salmona M and Diomede L (2018) Safety and toxicology of Magnolol and Honokiol. Planta Med 84, 1151–1164. 29925102 10.1055/a-0642-1966

[mol212645-bib-0026] Sengupta S , Nagalingam A , Muniraj N , Bonner MY , Mistriotis P , Afthinos A , Kuppusamy P , Lanoue D , Cho S , Korangath P *et al* (2017) Activation of tumor suppressor LKB1 by honokiol abrogates cancer stem‐like phenotype in breast cancer via inhibition of oncogenic Stat3. Oncogene 36, 5709–5721. 28581518 10.1038/onc.2017.164 PMC5793218

[mol212645-bib-0027] Shi P , Oh YT , Deng L , Zhang G , Qian G , Zhang S , Ren H , Wu G , Legendre B Jr , Anderson E *et al* (2017) Overcoming acquired resistance to AZD9291, a third‐generation EGFR inhibitor, through modulation of MEK/ERK‐dependent Bim and Mcl‐1 degradation. Clin Cancer Res 23, 6567–6579. 28765329 10.1158/1078-0432.CCR-17-1574 PMC5668147

[mol212645-bib-0028] Shi P , Oh YT , Zhang G , Yao W , Yue P , Li Y , Kanteti R , Riehm J , Salgia R , Owonikoko TK *et al* (2016) Met gene amplification and protein hyperactivation is a mechanism of resistance to both first and third generation EGFR inhibitors in lung cancer treatment. Cancer Lett 380, 494–504. 27450722 10.1016/j.canlet.2016.07.021

[mol212645-bib-0029] Song KA , Hosono Y , Turner C , Jacob S , Lochmann TL , Murakami Y , Patel NU , Ham J , Hu B , Powell KM *et al* (2018) Increased synthesis of MCL‐1 protein underlies initial survival of EGFR‐mutant lung cancer to EGFR inhibitors and provides a novel drug target. Clin Cancer Res 24, 5658–5672. 30087143 10.1158/1078-0432.CCR-18-0304

[mol212645-bib-0030] Sun SY , Yue P , Dawson MI , Shroot B , Michel S , Lamph WW , Heyman RA , Teng M , Chandraratna RA , Shudo K *et al* (1997) Differential effects of synthetic nuclear retinoid receptor‐selective retinoids on the growth of human non‐small cell lung carcinoma cells. Cancer Res 57, 4931–4939. 9354460

[mol212645-bib-0031] Tanimoto A , Takeuchi S , Arai S , Fukuda K , Yamada T , Roca X , Ong ST and Yano S (2017) Histone deacetylase 3 inhibition overcomes BIM deletion polymorphism‐mediated osimertinib resistance in EGFR‐mutant lung cancer. Clin Cancer Res 23, 3139–3149. 27986747 10.1158/1078-0432.CCR-16-2271

[mol212645-bib-0032] Tartarone A and Lerose R (2015) Clinical approaches to treat patients with non‐small cell lung cancer and epidermal growth factor receptor tyrosine kinase inhibitor acquired resistance. Ther Adv Respir Dis 9, 242–250. 26016841 10.1177/1753465815587820

[mol212645-bib-0033] Torre LA , Siegel RL and Jemal A (2016) Lung cancer statistics. Adv Exp Med Biol 893, 1–19. 26667336 10.1007/978-3-319-24223-1_1

[mol212645-bib-0034] Wang X , Duan X , Yang G , Zhang X , Deng L , Zheng H , Deng C , Wen J , Wang N , Peng C *et al* (2011) Honokiol crosses BBB and BCSFB, and inhibits brain tumor growth in rat 9L intracerebral gliosarcoma model and human U251 xenograft glioma model. PLoS ONE 6, e18490. 21559510 10.1371/journal.pone.0018490 PMC3084695

[mol212645-bib-0035] Woodbury A , Yu SP , Wei L and Garcia P (2013) Neuro‐modulating effects of honokiol: a review. Front Neurol 4, 130. 24062717 10.3389/fneur.2013.00130 PMC3769637

